# Response to road traffic accidents by Hashtroud Emergency Medical Services: a cross-sectional study 

**Published:** 2019-08

**Authors:** Safa Elmi, Mohammadreza Gholipour, Javad Babaie

**Affiliations:** ^*a*^Imam Hussein Hospital, Hashtroud, Tabriz University of Medical Sciences, Tabriz, Iran.; ^*b*^Faculty of Health ,Tabriz University of Medical Sciences Tabriz, Iran.; ^*c*^Department of Health Services Management, School of Management and Medical Informatics, Tabriz University of Medical Sciences Tabriz , Iran.

## Abstract

**Background:**

Road traffic accidents (RTAs) are among the most important causes of mortalities in Iran and worldwide. Pre-hospital emergency medical services (EMS) compromise a vital part of health services and have crucial role in response to RTAs. Purposes: The aim of present study is response to road traffic accidents by Hashtroud Emergency Medical Services in 2018, East Azerbaijan province.

**Methods:**

This cross-sectional study has investigated RTAs missions of Hashtroud EMS in 2018. Data were collected using registration database and archives in emergency service providing centers. Descriptive statistics and Microsoft Excel were utilized for data analysis.

**Results:**

Of 311 registered RTAs missions, urban missions were 112 cases and road accidents were 199. The average age of injured patients was 32.94. 79.6% were male and 25.1% were female. 81.3% of them resulted in transferring to the hospital and 10.7 % were treated in the scene while 8% of the missions were canceled.

**Conclusions:**

Importance of EMS in response to RTAs highlights the significance of proper and on-time services to prevent from disabilities, proper and fast transfer of patients by professional staff, and making systematic plans to promote medical services in terms of pre-hospital emergency services.

**Keywords:**

Road Traffic Accidents, Emergency medical services, Response

